# Hydrothermal ZnO Nanomaterials: Tailored Properties and Infinite Possibilities

**DOI:** 10.3390/nano15080609

**Published:** 2025-04-15

**Authors:** Muhammad Zamir Hossain, S. M. Abu Nayem, Md. Shah Alam, Md. Imran Islam, Gimyeong Seong, Al-Nakib Chowdhury

**Affiliations:** 1 Department of Chemistry, Jagannath University, Dhaka 1100, Bangladesh; m180301010@chem.jnu.ac.bd (S.M.A.N.); m170301033@chem.jnu.ac.bd (M.S.A.); b170301001@chem.jnu.ac.bd (M.I.I.); 2 Department of Environmental and Energy Engineering, The University of Suwon, 17, Wauan-gil, Bongdam-eup, Hwaseong-si 18323, Republic of Korea; 3 Department of Chemistry, Bangladesh University of Engineering and Technology, Dhaka 1000, Bangladesh; nakib@chem.buet.ac.bd

**Keywords:** ZnO, nanomaterial, hydrothermal synthesis, composite, photocatalysis

## Abstract

This review presents a comprehensive and precise summary of the hydrothermal synthesis and morphology control of zinc oxide (ZnO) nanomaterials, the advantages of hydrothermal synthesis, and the wide range of applications. ZnO nanomaterials have garnered significant attention in recent years for their diverse applications across various industries owing to their unique properties and versatility, with practical applications in healthcare, cosmetics, textiles, automotive, and other sectors. Specifically, the ability of ZnO-based nanomaterials to promote the production of reactive oxygen species, release of Zn^2+^ ions, and induce cell apoptosis makes them well-suited for bio-medicinal applications such as cancer treatment and microorganism control. Hydrothermal technique offers precise control over the synthesis of ZnO, metal/non-metal-doped ZnO, and related composites, enabling the tailoring of properties for specific applications. The significant feature of the hydrothermal technique is the use of water as a solvent, which is cheap, available, and environmentally benign. In the last section, we discussed the potential future direction of ZnO-based research.

## 1. Introduction

Due to their small sizes, nanomaterials (NMs) exhibit a high surface area-to-volume ratio, making their behavior distinct from their bulk counterparts while often displaying advantageous physicochemical properties [1,2,3,4,5,6]. Among different NMs, ZnO is a group II-VI semiconductor (bandgap 3.37 eV), strong Zn–O bonding (60 meV), excellent piezoelectric and pyroelectric properties, mechanical robustness, biocompatibility, high stability, and broadband light absorption. These properties make ZnO highly suitable for various applications, including gas sensing, energy conversion and storage, photocatalysis, bacterial management, (opto)electronics, lasers, and more [1,7,8,9,10,11,12,13,14,15,16,17,18,19,20].

In the past, ZnO was produced using the so-called French process and employed as a pigment for oil paints and watercolors [21,22,23]. Later, it assumed a vital role in tire manufacturing as an activator of vulcanization accelerators. Currently, the rubber industry consumes 50–60% of the ZnO produced globally, while the remainder is used in the production of ceramics, concrete, skin ointments, sunscreen lotions, food supplements, and pigments [24,25,26,27,28]. High-purity ZnO is also utilized in the production of semiconductors with nonlinear current–voltage characteristics. Currently, high-purity ZnO is produced from metallic Zn extracted from suitable ores, followed by thermal processing and doping to enhance its photoconductive and semiconductor performance [26,29,30,31,32,33,34,35].

Interestingly, nanoscale ZnO has a higher surface-to-volume ratio than their bulk counterpart. One of the key features of ZnO NMs is antibacterial activity, as it can generate reactive oxygen species (ROS). This property makes them effective agents for inhibiting bacterial growth and preventing microbial contamination in various environments [36,37,38]. Furthermore, ZnO NMs exhibit exceptional photocatalytic properties in dye degradation and removing pesticides from water sources [39,40,41].

In addition, ZnO nanostructures play a pivotal role in advancing renewable energy technologies. For instance, they are employed in photocatalytic water-splitting processes to produce clean hydrogen fuel from water. Additionally, ZnO nanoparticles (NPs) are utilized as electron transport layers in solar cells, enhancing their efficiency by improving light absorption and charge separation within the devices [42,43,44].

Moreover, when dispersed in fluids, ZnO NPs demonstrate superior thermal conductivity, making them ideal candidates for nanofluid applications aimed at efficient heat transfer across various industrial and consumer systems [45,46].

**Figure 1:**
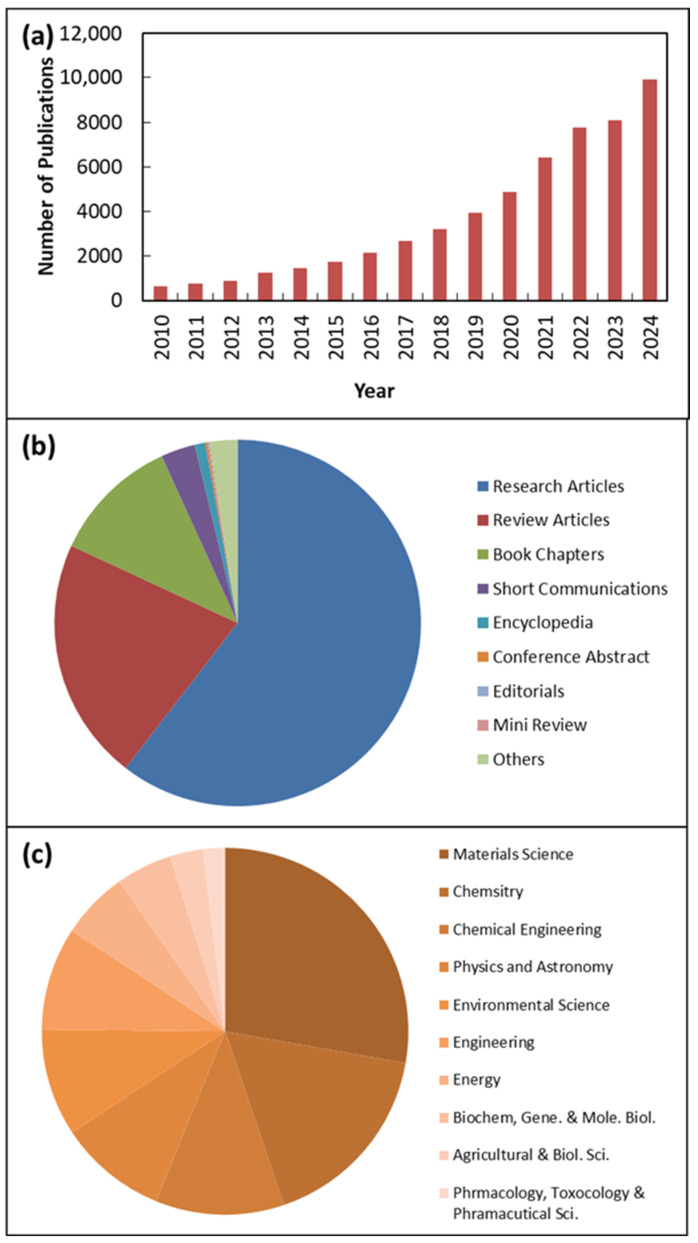
Publications identified via keywords “ZnO nanomaterials” from 2011 to 2024 according to sciencedirect.com. (**a**) Yearly number of publications between 2011 and 2024; (**b**) publications/article types and (**c**) subject area of published documents.

Research on ZnO NMs is gaining increasing popularity. The growing interest in ZnO NMs research between 2010 and 2024 is illustrated in Figure 1 according to sciencedirect.com, which shows relevant publications using the keyword “ZnO nanomaterials,” clearly demonstrating how research on ZnO NMs has become more prevalent in recent years. This provides clear evidence that research on ZnO NMs is steadily gaining attention and being actively pursued. Additionally, Figure 1 includes the subject areas and types of publications related to ZnO NMs. It is important to note that the two main fields of study for ZnO NMs are materials science and chemistry, chemical engineering, physics, and astronomy, as shown in Figure 1. Furthermore, among the published documents, 64% were research articles, while 18% were review articles.

An important area of materials science is the development and exploration of material synthesis techniques that are eco-friendly, user-friendly, cost-effective, and efficient. ZnO can be synthesized through various methods, each with its advantages and disadvantages. In chemical vapor deposition (CVD) [47,48,49], ZnO is grown by the reaction of a vapor-phase zinc precursor with oxygen. This method allows for precise control over the growth conditions and the resulting film properties. The sol-gel method involves the hydrolysis and condensation of metal precursors (such as zinc acetate or zinc nitrate) in a solvent to form a gel, which is then dried and calcined to obtain ZnO NPs or thin films [50,51]. Sputtering, another physical vapor deposition technique, deposits ZnO thin films by bombarding a zinc target with high-energy ions in a vacuum chamber, causing zinc atoms to eject and condense on a substrate to form a thin film [52,53]. In electrochemical deposition techniques, ZnO films or nanostructures can be electrodeposited onto a conductive substrate by applying an electric current to a zinc salt solution [54,55]. In spray pyrolysis, a precursor solution containing zinc salts is sprayed onto a heated substrate, where it decomposes to form ZnO [56,57,58]. With template-assisted synthesis, ZnO nanostructures with controlled morphology can be synthesized by using templates, such as porous membranes or self-assembled monolayers, to guide the growth of ZnO [59].

However, hydrothermal synthesis is superior to conventional methods as it largely avoids the particle manipulation-related drawbacks of solid-phase synthesis [60,61,62,63,64,65]. Moreover, hydrothermal methods offer advantages such as high product purity, short reaction time, precise control over particle shape and size [61,65,66,67,68,69]. Hydrothermal processes use high-temperature water as the reaction medium and can be employed to produce various types of materials (e.g., single crystals and ultrafine or less agglomerated crystalline ceramic powders). A notable feature of the hydrothermal technique is the use of water as a solvent, which is cheap, available, and environmentally benign. Using hydrothermal routes, NMs with different structures like nanospheres, nanorods, nanocubes, nanosheets, nanowires, and nanotubes are possible to synthesize. Given their versatility and growing importance, hydrothermal techniques have significantly advanced over time and are now widely utilized across fields such as materials science, earth science, metallurgy, physics, chemistry, biology, and others.

Hydrothermal synthesis primarily occurs through two main pathways: dissolution–precipitation and dissolution–crystallization [70]. Initially, the starting material dissolves to form ions or ionic groups, and as the temperature increases, solubility rises. Once the concentration reaches a certain level, particle nucleation and growth take place, resulting in the formation of the final product. Compared to conventional reactions that typically occur at high temperatures, hydrothermal synthesis, conducted under high pressure, allows reactions to be effectively induced at lower temperatures. Additionally, during hydrothermal synthesis, the escape of volatile reactants is prevented, and the chemical composition can be maintained stably.

Hydrothermal reactions are influenced by various factors, such as the concentration of starting materials (typically a mineralizer and metal salts or hydroxides), reaction temperature, reaction time, pH of the solution and the solvent. The starting material determines the types of possible reactions, and choosing the appropriate starting material is crucial for producing high-quality particles. Mineralizers play a vital role in transforming unstable solids into more stable forms through dissolution-precipitation or crystallization processes, and they have a significant impact on the final product’s chemical composition. Additionally, the type of mineralizer (e.g., hydroxides, carbonates, halides) affects the particle size and shape [61,71,72,73,74].

This review aims to comprehensively cover the latest research trends in the hydrothermal synthesis of ZnO-based NMs and their potential industrial applications. By providing an in-depth understanding of these areas, it offers crucial guidance for future research and commercialization, serving as an important reference for researchers and industry professionals to effectively harness the potential of nanotechnology.

## 2. Features and Advantages of Hydrothermal Synthesis

‘Hydro’ means water and ‘therm’ means temperature. Therefore, hydrothermal synthesis refers to a method for synthesizing nanomaterials (NMs) by applying heat to aqueous solutions containing precursors. High-temperature and pressure conditions in hydrothermal synthesis enable the formation of various NMs with desirable properties, such as high crystallinity, fine particle size, and improved homogeneity. These conditions increase the solubility of precursors and promote the formation of supersaturated solutions, which facilitates uniform nucleation throughout the medium. As a result, homogeneous particle growth is achieved. Moreover, elevated temperatures enhance atomic mobility and diffusion rates, allowing for defect-free crystal formation and leading to improved crystallinity. The pressurized aqueous environment also enables reactions to proceed above water’s boiling point while maintaining the liquid phase, providing stable conditions for the growth of well-faceted crystals with controlled size and morphology.

Hydrothermal synthesis is a simple, environmentally friendly process that requires minimal post-processing, making it ideal for large-scale material preparation. It utilizes solution- and reaction-based methodologies, where temperature and pressure govern the final material morphology [2,68,75,76]. Compared to other methods, hydrothermal synthesis enhances NM stability at high temperatures and minimizes material loss, which is especially important for compounds with high vapor pressures. The product composition can also be precisely tuned via liquid-phase or multiphase reactions. Crystal size and shape vary depending on the initial mixture composition, as well as the temperature and pressure conditions applied during synthesis [27,77,78,79].

**Figure 2:**
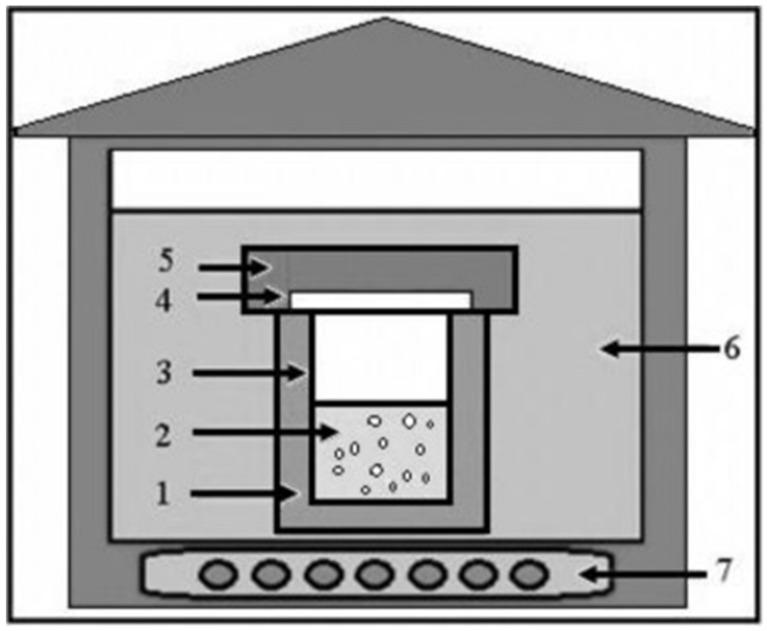
General outline of the equipment used for hydrothermal synthesis. (1) Stainless-steel autoclave, (2) reagent solution, (3) Teflon lining, (4) Teflon lid, (5) stainless-steel lid, (6) heat transfer medium (water or air), (7) electrical heater. Reprinted with permission from [71], 2016, Elsevier.

The size and morphology of ZnO NMs are critical in determining their structural and physicochemical properties, which, in turn, influence their performance in various industrial applications. To optimize these properties, precise control over size and shape is essential [29]. However, achieving this level of control involves optimization of several factors, including temperature, precursor concentration, pH, solvent selection, and the use of polar/non-polar surface modifiers and stabilizing agents. General hydrothermal synthesis pathways are illustrated in Figure 2.

## 3. Advanced Hydrothermal Synthesis of ZnO-Based Nanomaterials

### 3.1. Morphology and Size Control

It is well-documented that the morphology and size of ZnO NPs synthesized via hydrothermal method can be precisely controlled by manipulating several key parameters. Hosseinian et al. investigated the effect of pH on the synthesis of ZnO nanostructures and demonstrated that the shape and size of these structures were strongly influenced by the pH of the reaction solution [80]. Specifically, increasing the pH resulted in the shortening of both the length and diameter of the nanostructures.

ZnO nanostructures with four distinct morphologies of NPs, nanorods (NRs), mixtures of NPs and NRs, and nanoflowers formed by the aggregation of NPs and NRs were synthesized under mild conditions by adding NaF and NaOH with the assistance of hexamethylenetetramine (HMTA) [81]. HMTA produces NH_4_^+^ and OH^−^ which are responsible for NPs formation. ZnO NRs were synthesized in the presence of NaF as F^−^ hinders the NPs’ growth. However, the introduction of a small quantity of NaOH led to the transformation of the NRs into a mixture of NPs and NRs as OH^−^ hinders the activity of NRs growth. At high concentrations of NaOH, chemical equilibrium shifted in a way where NH_3_ decomposition decreased, hence ZnO nanoflowers composed of aggregated NPs and NRs were obtained.

Saranya et al. prepared ZnO nanostructures using equimolar amounts of HMTA and Zn(NO_3_)_2_ at 60 °C, 90 °C, and 120 °C [82]. They found that slower nucleation at lower temperatures resulted in the formation of NRs. The NRs formed at 90 °C had diameters of 200–500 nm and exhibited random orientation. At 120 °C, supersaturation and accelerated nucleation lead to the formation of rice-shaped structures with diameters of 2–4 μm. In the study by Ozturk et al., ZnO NRs were produced on a seed layer using an equimolar mixture of HMTA and Zn(NO_3_)_2_·6H_2_O [83]. The size of the ZnO NRs was controlled by adjusting the precursor concentration, which had a much greater effect on the NR diameter than the NR length. In another experiment, ZnO NRs were synthesized from a 1:2 (mol/mol) mixture of NaOH and Zn(CH_3_COO)_2_ at 170 °C for 12 h [84].

Additionally, another study produced ZnO NPs with a flower-like morphology using NaOH and Zn(NO_3_)_2_·6H_2_O as precursors [85]. Flower-shaped ZnO NPs were synthesized at 100 °C and 125 °C, while spherical NPs were obtained at 150 °C. With increasing reaction time, the grain size increased [86].

**Figure 3:**
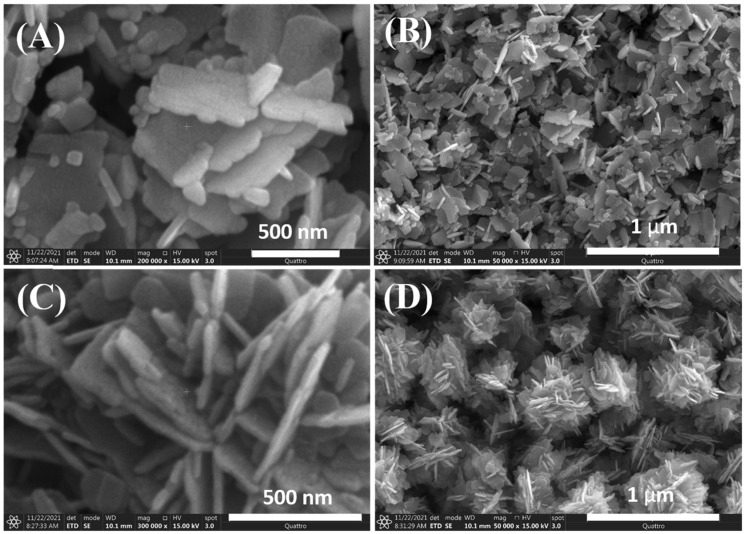
Variable magnification field emission scanning electron microscopy images of ZnO NFs synthesized (**A**,**B**) without and (**C**,**D**) with jute stick extract. Reprinted with permission from [87], 2022, Elsevier.

The shape of NPs can be adjusted by using biological or plant extracts. In our previous study, we used jute stick extract as a substrate for ZnO nanoflower (NFs) growth at 170 °C for 5 h [87]. The resulting ZnO NFs exhibited a collective flower-shaped structure composed of NFs (Figure 3). A comparison of the ZnO NFs produced with and without jute extract suggests that the change in NFs shape is due to the electrostatic interaction between Zn^2+^ ions and the polar groups of biomolecules in the extract.

### 3.2. Doping Effects on ZnO NPs: Metal and Non-Metal Modifications

The physicochemical characteristics of ZnO NPs can be altered by doping. In particular, the dielectric properties of ZnO NPs can be tuned by doping because of the presence of inherent defects, such as Zn vacancies, grain boundary modulation, dangling bonds, Zn interstitials, and oxygen vacancies. The development of NPs and the formation of native defects therein may be significantly influenced by selective dopants, depending on their atomic radii and charge valence states [69].

**Figure 4:**
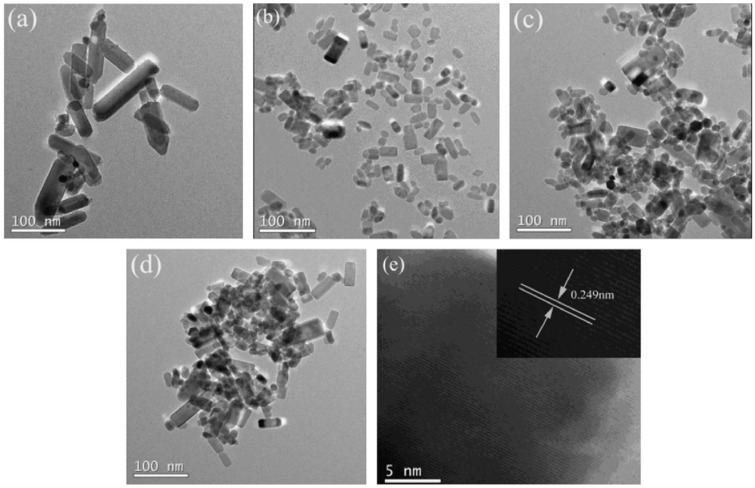
(**a**–**e**) HRTEM micrographs of Fe- and Ni-doped ZnO samples. Reprinted with permission from [88], 2014, Elsevier.

Fang et al. synthesized Cu-doped ZnO NPs via a microwave-assisted method at 100 °C for 20 min [73]. The material produced displayed ferromagnetic behaviour due to the interaction between Cu^2^⁺ and native defects, such as oxygen vacancies in ZnO. The introduction of Cu intensified the ferromagnetic signal, whereas pure ZnO remained diamagnetic. The doping micrographs are shown in Figure 4.

In another study, a ZnO-Ag composite was synthesized by adding AgNO_3_ to a solution of Zn(CH_3_COO)_2_·2H_2_O and NaOH [89]. After heating the mixture in a Teflon-lined stainless-steel reactor at 170 °C, a ZnO-Ag composite was formed. The doping of Ag introduced unique optical and electrical properties to the ZnO matrix. Similarly, Zn_0.95_Fe_0.05−*x*_Ni*_x_*O (where *x* = 0, 0.02, and 0.05) diluted magnetic semiconductors were produced by a hydrothermal method [88]. This doping introduced excellent magnetic properties for applications in spintronics.

Boron doping in ZnO was achieved using Zn(CH_3_COO)_2_·2H_2_O, HMTA, and H_3_BO_3_ [90]. The reaction was carried out in a Teflon-lined reactor at 95 °C, and the resulting powder was calcined at 750 °C. The B-doped ZnO exhibited altered optical properties and reduced bandgaps due to the introduction of B at the Zn site. The extent of doping was adjusted by changing the B concentration from *x* = 0.0 to *x* = 0.11 in the formula of Zn_1−*x*_B*_x_*O.

### 3.3. ZnO-Based Nanocomposites

Graphene/ZnO nanocomposites were synthesized at 80 and 90 °C to study the effects of temperature on their properties [91]. A graphene oxide dispersion was prepared then the dispersion was pH-adjusted to 4 and supplemented with 0.5 M Zn(CH_3_COO)_2_·6H_2_O under continuous stirring for 15 min. To enhance the conversion of Zn(OH)_2_, aqueous NH_3_ was added to adjust the pH to 11, and the mixture was stirred for 30 min. After hydrothermal treatment at 80 °C or 90° C for 10 h, the graphene/ZnO composites were oven-dried overnight at 70 °C to get the final product.

In a separate study, CuO/ZnO nanocomposites with varying phase ratios were synthesized in one step method [92]. With increasing CuO content, the nanosheets collapsed, and the number of smaller sheets increased. The observed room-temperature ferromagnetism at the interface between diamagnetic ZnO and antiferromagnetic CuO could be fine-tuned by adjusting the ZnO/CuO ratio.

A simple hydrothermal synthesis method has been presented for the preparation of a new ternary α-Fe_2_O_3_–ZnO–Au nanocomposite by surface coating ZnO and Au NPs onto α-Fe_2_O_3_ NRs [93]. This method demonstrates the feasibility of synthesizing ternary nanocomposites with distinct materials. The nanocomposite exhibited high sensitivity/responses to 100-ppm n-butanol and acetone at an optimal operating temperature of 225 °C.

Li et al. developed a polypyrrole/ZnO nanohybrid through a hydrothermal treatment method [94]. Polyvinyl alcohol containing zinc acetate was electrospun into nanofibers, which were then hydrothermally treated at temperatures below 150 °C to form nanostructured ZnO, on the surface of Au electrodes. Polypyrrole was then deposited onto the ZnO surface via vapor-phase polymerization. The composite exhibits high-performance characteristics as gas sensors, providing highly sensitive, repeatable, and selective responses to NH_3_ over a wide concentration range (0.5–200 ppm) at room temperature. ZnO-based nanocomposites consisting of ZnO-xTiO_2_, and ZnO-xCeO_2_ (x  =  5 wt.% or 10 wt.%) prepared by the sol-gel method is used for hydrogen release. The materials displayed a hydrogen generation rate of 3000 mL·min^−1^·gcat^−1^ [95].

### 3.4. ZnO-Based Core-Shell NPs

Core-shell NPs have garnered significant interest due to their customizable compositions and structures, making them suitable base materials for various device applications. Moreover, the synergistic interactions between the core and shell can introduce advantageous novel properties [96,97,98]. One study reported the synthesis of Fe_3_O_4_@ZnO core–shell NPs using Azadirachta indica leaves in a two-step process combining phyto and hydrothermal techniques [99]. In the first step, highly magnetic Fe_3_O_4_ NPs were synthesized using Azadirachta indica leaf extract. In the second step, the resulting Fe_3_O_4_ NPs were used as cores for a hydrothermal process to coat ZnO shells.

TiO_2_/ZnO core-shell NPs were also synthesized using a two-stage hydrothermal method [74]. In the first stage, ZnO NPs were prepared by dissolving zinc nitrate hexahydrate (Zn(NO₃)₂·6H₂O) in distilled water and adjusting the pH to ~9. The solution was vigorously stirred and heated in a Teflon-lined autoclave at 220 °C for 5 h to get ZnO NPs. In the second stage, ZnO NPs were coated with TiO_2_ by preparing a TiO_2_ suspension with polyvinyl alcohol, ethyl alcohol, and titanium isopropoxide. 

Mesoporous ZnO-TiO_2_ hollow spheres were synthesized with the assistance of hydrothermal treatment, using carbohydrate as a template [100]. Carbohydrates underwent thermal processing in the reactor to partially form the hollow spheres. This intermediate product was then functionalized by attaching -SO₃H groups to the mesopore walls using chlorosulfonic acid in chloroform.

**Figure 5:**
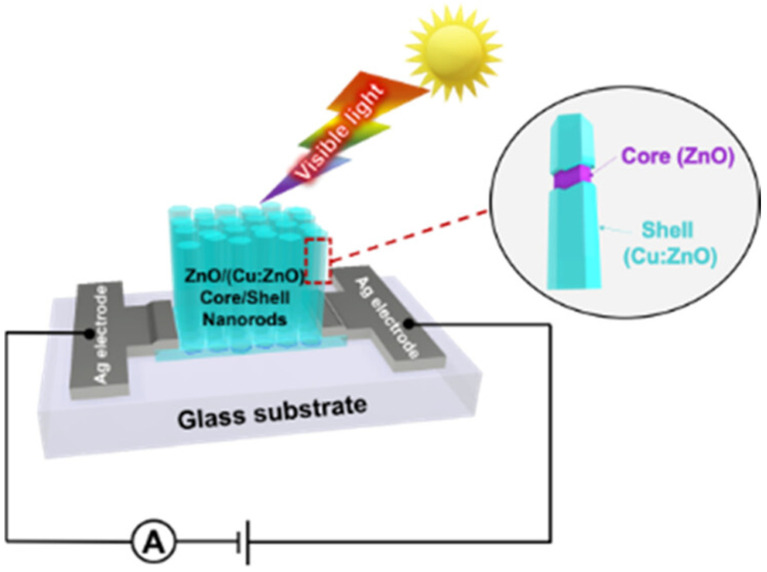
Schematic of enhancing ZnO-based photodetectors’ performance via the formation of ZnO/(Cu:ZnO) core–shell nanorods. Reprinted from [101].

Besides doping, architecting ZnO NMs to extend their application is still attractive for developing low-cost optoelectronic devices. Thereby, a study introduced the innovation of ZnO NR-based photodetectors by combining two techniques, which are Cu doping and core/shell structuring. Particularly, the active material (ZnO/(Cu-doped ZnO) core/shell NRs) was synthesized by a low-cost and easy fabrication process. The device using the developed core/shell NRs exhibited the maximum responsivity of ca. 4.5 mA W^–1^ at 395 nm light exposure, which is nearly 180% higher than that of a device based on Cu-doped ZnO NRs (formed with only the Cu-doping technique). This enhancement responsivity is attributed to the improved charge transport at the interface of the core/shell ZnO/(Cu-doped ZnO) NRs (Figure 5), which pertains to a process that reduces the electron–hole recombination probability, resulting in an improvement in the efficiency of the photodetectors [101].

Table 1 summarizes different synthesis conditions of the precursor type, temperature, pH, and reaction time that influence the morphology of ZnO nanostructures.

## 4. Applications of ZnO-Based Nanomaterials

### 4.1. Sensing

ZnO, an *n*-type II–IV semiconductor, holds significant potential for gas sensing applications due to its high chemical stability, excellent optical properties, nontoxicity, ease of doping, low cost, high surface-to-volume ratio, and remarkable sensitivity even at low temperatures [102,103,104,105]. Additionally, efficient enzyme management and uniform matrix distribution are critical in developing biosensors utilizing ZnO. The superior electrical and optical properties of nanostructures, resulting from electron and phonon confinement, provide an effective platform for enzyme immobilization. ZnO-based NMs create an optimal microenvironment and offer a large surface area to increase enzyme loading while preserving enzymatic bioactivity. These exceptional properties, combined with high performance and ease of fabrication, have made ZnO-based NMs widely applicable in the field of electrochemical (bio)sensors [26,29,30,31,32,33,34,35].

**Figure 6:**
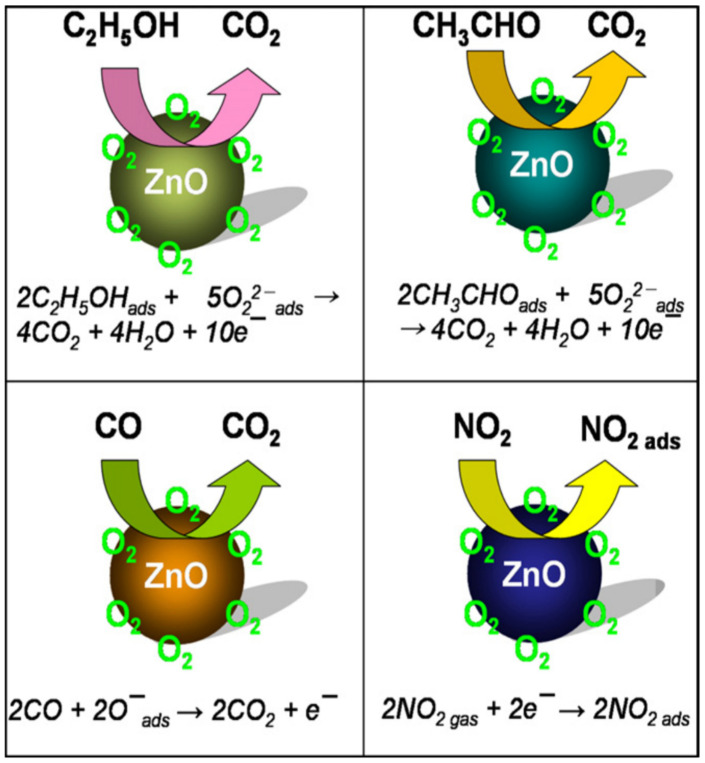
Schematic gas sensing mechanism of ZnO NPs. Reprinted with permission from [30], 2012, Elsevier.

Fibre-optic-based localised surface plasmon resonance (FO-LSPR) sensors with exceptional sensitivity were developed using three-dimensional (3D) nanostructures composed of ZnO nanowires and Au NPs [106]. The 3D nanostructures, fabricated by synthesizing ZnO nanowires on the cross-sections of optical fibers and decorating them with Au NPs, enabled significant advancements in biosensor performance. Compared to two-dimensional (2D) FO-LSPR sensors, the 3D nanowire structures provided distinct advantages, such as a higher density of Au NPs per unit area for increased light focus, a porous architecture facilitating efficient target molecule binding, and unique structural characteristics that amplified overall performance. In another report, ZnO nanowire arrays prepared by the hydrothermal method were used to detect H_2_ and CO at room temperature [107]. A scheme of the gas sensing mechanism is shown in Figure 6.

**Figure 7:**
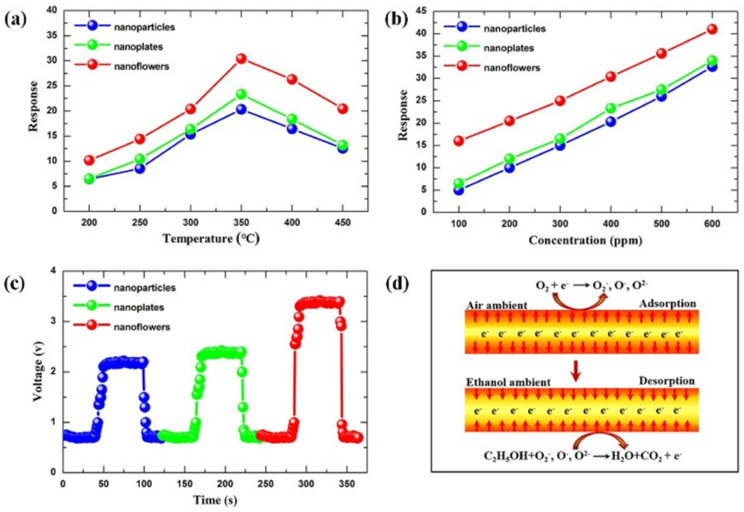
(**a**) Response of the sensors made of the ZnO with different nanostructures exposed to 400 ppm ethanol at various temperatures. (**b**) Response of the three sensors under different ethanol concentrations at 350 °C. (**c**) Response–recovery curve of the three sensors at 350 °C towards 400 ppm ethanol. (**d**) The mechanism of the sensor. Reprinted with the permission from [7], 2018, Elsevier.

Hierarchical Sn_3_O_4_ nanoflowers decorated with ZnO NPs were used to fabricate a gas sensor that exhibited excellent performance for selective and repeatable formaldehyde detection at a low operating temperature of 180 °C, with a detection limit of 1 ppm [108]. The sensor showed a fast response time of 5 s for 100 ppm formaldehyde, with the ZnO/Sn_3_O_4_ heterojunction enhancing gas-sensing performance as electron accumulation on the surface due to the different work function of ZnO and Sn_3_O_4_. In an oxygen-containing atmosphere, oxygen molecules adhered to the sensor surface, trapping free electrons and forming O_2_^−^, O^−^, or O_2_^−^ species, thus increasing the sensor’s barrier height and resistance. Formaldehyde then reacted with the surface-adsorbed oxygen species, releasing electrons and decreasing the sensor’s resistance. Meanwhile, ZnO NPs, nanoplates, and NFs were successfully synthesized via a simple hydrothermal method, and their gas-sensing properties to ethanol were also investigated [7]. Among the different nanostructures, nanoflowers assembled from nanoplates exhibited the highest performance, attributed to their hierarchical architecture providing a large surface area and abundant spaces for gas diffusion (Figure 7). Furthermore, it was surprisingly found that the concentration of surfactant (cetyltrimethylammonium bromide) played a crucial role in determining the final morphology of the hierarchical NFs.

Al-doped ZnO NPs were used to detect volatile organic compounds (VOCs) such as acetaldehyde, toluene, and benzene [109]. At the optimal operating temperature of 500 °C, the highest sensing response was observed for 10 ppm acetaldehyde, which was 173 and 125 times higher than those observed for toluene and benzene, respectively. The elevated sensitivity to acetaldehyde was attributed to its larger dipole moment, and the enhanced performance of Al-doped ZnO NPs was linked to the increased specific surface area, conductivity, and oxygen vacancy content due to doping. The increased oxygen vacancy content provided more adsorption sites for acetaldehyde on the NP surface, and the larger optical bandgap of Al-ZnO generated more ionized oxygen ions reacting with acetaldehyde. Similarly, Li et al. synthesized α-MoO_3_/0D ZnO nanocomposites and achieved highly sensitive ethanol detection at a relatively low temperature of 250 °C [110]. The high ethanol sensing performance was attributed to the synergistic effect and the creation of a Schottky barrier and additional depletion layers between the two materials. Ethanol reacted with the abundant chemisorbed O_δ−_ atoms on both α-MoO_3_ and ZnO surfaces, enhancing the release of trapped electrons compared to a pure α-MoO_3_ sensor.

### 4.2. Photocatalysis

ZnO NPs can absorb photons and induce the formation of electron-hole pairs on the catalyst surface through oxidation and reduction reactions, enabling applications such as organic pollutant degradation, water splitting, microorganism control, photocatalytic conversion, and organic synthesis [111,112,113,114,115,116,117].

#### 4.2.1. Dye Degradation

Industrial wastewater and waste generated by human activities increase the concentration of pollutants in water bodies, significantly impacting the global ecosystem and economic growth. Advanced photocatalytic oxidation processes, which effectively remove organic pollutants and heavy metals, have made ZnO a highly promising choice for water treatment. ZnO is widely used due to its excellent chemical stability, low toxicity, strong oxidation capacity, outstanding photocatalytic performance, and easy availability [118,119,120].

ZnO NPs promote the photocatalytic degradation of various organic pollutants, including methylene blue (MB), aniline, rhodamine B (RhB), four chlorophenols, Congo red, Brilliant Green, Malachite Green, Direct Blue 15, Brilliant Yellow, and phenazopyridine [121,122,123,124,125,126,127,128,129,130]. The photocatalytic decomposition process of organic pollutants over ZnO NPs involves the generation of ROS (e.g., •OH and O₂^−^•) and holes (h^+^) in the reaction medium. These ROS engage in redox reactions with organic pollutants adsorbed on NPs’ surface, converting them into non-toxic and simple inorganic compounds. The photocatalytic efficiency of ZnO is significantly influenced by its surface area, size, morphology, and crystal defects [131,132].

Kumar et al. synthesized ZnO-MoS_2_-rGO (reduced graphene oxide) heterostructures and demonstrated that these composites enhanced the photocatalytic degradation of MB. The composite improved UV and visible light absorption, promoting charge carrier generation and rapid charge transfer, which minimized recombination and effectively supported pollutant degradation [133]. The large surface area of rGO further helped adsorb MB, contributing to the degradation process. Similarly, Mn-doped ZnO NPs attached to rGO sheets formed a ternary heterostructure, exhibiting superior photocatalytic performance for RhB and Congo red degradation under sunlight. This was attributed to enhanced sunlight absorption, increased organic compound adsorption, and effective charge separation [134].

**Figure 8:**
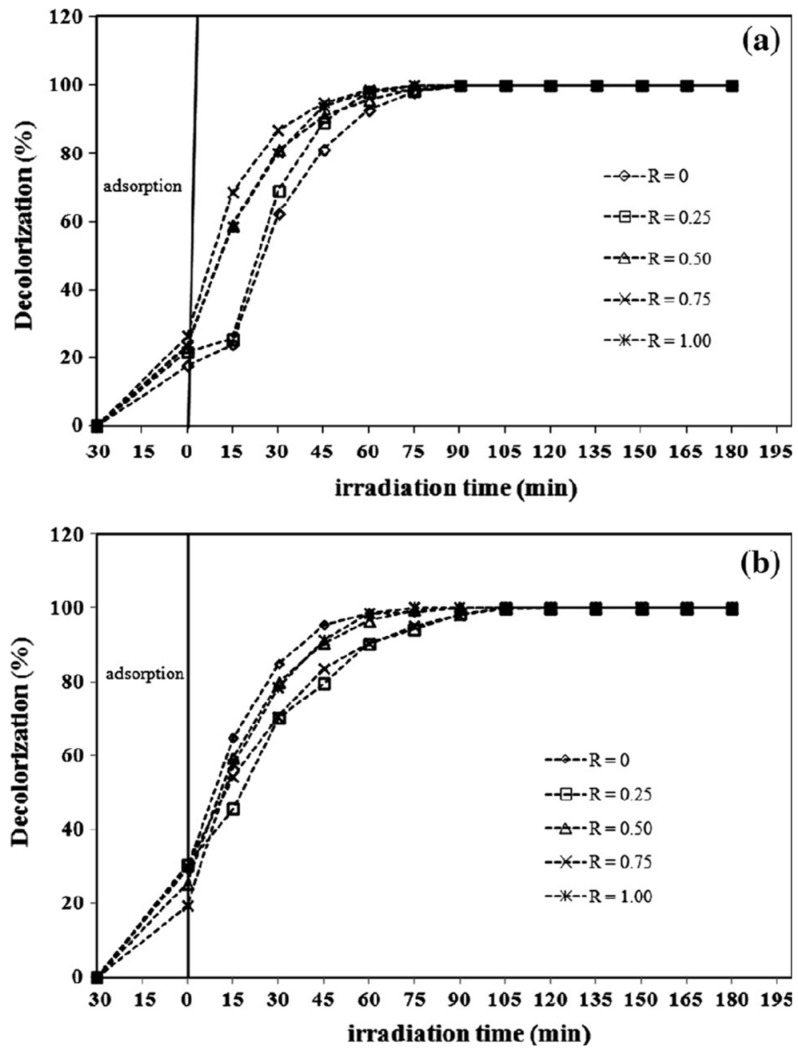
Photocatalytic MB degradation by ZnO nanostructures prepared at different molar ration of PEO19-b-PPO_3_ (*R*, 0–1.00) using alkaline solutions of (**a**) KOH and (**b**) CO(NH_2_)_2_. Reprinted with permission from [135], 2016, Elsevier.

The photocatalytic efficiency of ZnO has been enhanced through various structural modifications as shown in Figure 8. The dependence of crystallite size, particle shape, and oxygen vacancy on the optical properties to facilitate photocatalytic degradation in ZnO nanostructures was investigated. ZnO nanoplatelets and mesh-like ZnO lamellae were synthesized using a hydrothermal method [135]. The band tail energy of ZnO nanostructures had a greater effect on the bandgap energy than the crystallite size. The ZnO nanoplatelets exhibited better photocatalytic degradation performance for MB compared to the mesh-like ZnO lamellae, attributed to the migration of photoelectrons and holes to the (0001) and (000−1) planes, respectively, under the internal electric field. ZnO synthesized with amino acids like glutamine, histidine, and glycine in a hydrothermal process showed remarkable activity for RhB degradation due to its unique hierarchical structure and high oxygen defect concentration [136]. Similarly, ZnO NRs synthesized from Zn powder using acetylacetone exhibited improved photocatalytic efficiency after annealing, with increased bandgap and oxygen defect concentration [137].

**Figure 9:**
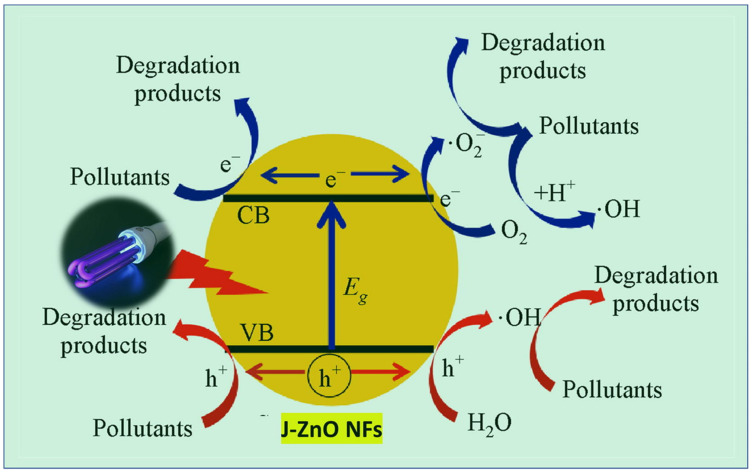
Mechanism of MB photodegradation catalyzed by J–ZnO NFs. Reprinted with permission from [87], 2022, Elsevier.

In another experiment, ZnO NFs synthesized with jute (J-ZnO NFs) demonstrated a significant increase in the photocatalytic degradation efficiency of MB under UV light (Figure 9) [87]. After 8 h, the degradation efficiency reached 98%, surpassing the performance of bare ZnO. Moreover, ZnO-loaded graphene nanostructures exhibited excellent photocatalytic activity due to their reduced bandgap and the synergistic effects between the ZnO and graphene components. When coated onto glass substrates, these nanohybrids showed excellent transparency and superhydrophilicity, making them suitable for self-cleaning applications [138].

Finally, ZnO NPs synthesized from aqueous NH_3_ and ZnCl_2_ exhibited different morphologies depending on the pH levels and demonstrated high stability. These NPs achieved a degradation efficiency of 94% for RhB after five cycles, with the highest degradation efficiency observed at pH 9 [139].

#### 4.2.2. Water Splitting

The pursuit of zero-carbon energy and the rising global demand for sustainable and eco-friendly energy sources have driven significant attention toward hydrogen production through electrochemical water splitting [140]. This method offers a promising route to achieving cleaner energy sources, especially when employing semiconducting materials such as ZnO.

In electrochemical water splitting, ZnO NPs are used in an aqueous electrolyte with a specified potential difference to facilitate photocatalytic water splitting. Charge transfer occurs between the electrode and electrolyte until an equilibrium Fermi level is reached. This electron flow leads to the accumulation of positive charge in the space–charge region and upward bending of the band edges. As a result, the photoexcited electrons flow unidirectionally toward the photocathode, and the holes on the ZnO NPs serve as active sites for water oxidation [111].

ZnO NRs have been successfully grown on SiO_2_/Si and indium tin oxide (ITO) substrates using the hydrothermal method. The diameters of these NRs ranged from 45 to 275 nm. These smaller diameter NRs showed enhanced photoconversion efficiency due to increased light absorption, confirmed by finite-difference time-domain simulations [141]. Additionally, ZnO NRs doped with boron were synthesized using the hydrothermal method on ITO substrates. Boron doping enhanced the photoinduced properties of ZnO NRs compared to those of the undoped NRs, making them more suitable as photoelectrodes in photoelectrochemical (PEC) cells. This enhancement was due to more effective charge separation and reduced recombination probability resulting from boron doping. Also, boron doping increases the conductivity and electron density on the conduction band [142].

ZnO NR arrays (NRAs) grown on Zn foil in a dual-alkaline solution have also been employed for photoelectrochemical water splitting [143]. The ZnO-ZnS photoanode obtained after 2 h of sulphidation exhibited a photocurrent density of 0.073 mA·cm^−2^ at 0 V vs. Ag/AgCl and a photoconversion efficiency of 0.034% at 0.63 V vs. RHE under 100 mW·cm^−2^ illumination. These results represent approximately 1.35- and 1.42-times greater performance than that of bare ZnO NRAs. N-doped ZnO NRAs were synthesized from a Zn-amine complex solution and annealed at ambient pressure. Electrochemical Mott-Schottky analysis suggested that N doping decreased carrier density, which could offer significant benefits for solar-powered water splitting [144].

In another study, nucleation-controlled, vertically oriented ZnO NRAs were fabricated on a conductive substrate for use in photoelectrochemical cells. These ZnO NRs, oriented along the c-axis and synthesized via the hydrothermal method, demonstrated enhanced PEC performance when ammonia was added to the growth solution. Ammonia improved NR development along the (002) direction by preventing precipitation of Zn(OH)_2_, leading to better performance due to the increased surface area, reduced charge recombination, and faster electron transit under illumination [145].

Further studies on the composition of (Zn_1−*x*_Co*_x_*)O nanowires (NWs), with *x* = 0, 0.05, and 0.1, showed improved solar-to-hydrogen efficiency (SHE) for (Zn_0.95_Co_0.05_)O NWs, which were selected for nitrogen co-doping. These nanostructured (Zn_1−*x*_Co*_x_*)O:N NWs, which were vertically aligned, exhibited significantly enhanced PEC performance due to improvements in light absorption, carrier density, and electrochemical charge transfer rates originating from doping of Co and N, which reduces the bandgap of ZnO [146].

#### 4.2.3. Pesticide Removal

The photodegradation efficiency of ZnO for pesticides (monocrotophos) was significantly improved by narrowing its bandgap and enhancing charge carrier separation through Cu doping, resulting in better visible light absorption. This led to increased charge carrier generation and improved pesticide mineralization under visible light [147]. Similarly, CuS quantum dots (QDs) were hybridized with ZnO, forming a nanocomposite (CuS QD@ZnO) that exhibited enhanced crystallinity, light absorption, surface area, and electron-hole pair separation. The decrease in the bandgap after hybridizing with CuS QD increases the pesticides’ degradation performance. The inhibition of electron-hole recombination at the interfacial heterojunction further enhanced photocatalytic performance, particularly in the degradation of the emerging pollutant, 2,4,5-T, with 3 wt% QD loading showing optimal results [148].

In another approach, ZnO NPs were synthesized with hollow pitchfork morphology, increasing the surface area and adsorption sites for more efficient photocatalytic decomposition of malathion. This improvement was attributed to the synergistic effect of photo-piezo catalysis driven by photo-vibration energy [149]. The incorporation of reduced graphene oxide (rGO) into a ZnO-rGO nanocomposite was found to decelerate electron-hole recombination and enhance carrier transport, boosting the photocatalytic degradation of dimethoate. More specifically, the incorporation of rGO leads to increased separation of electrons and holes, which enhance the photodegradation [150].

**Figure 10:**
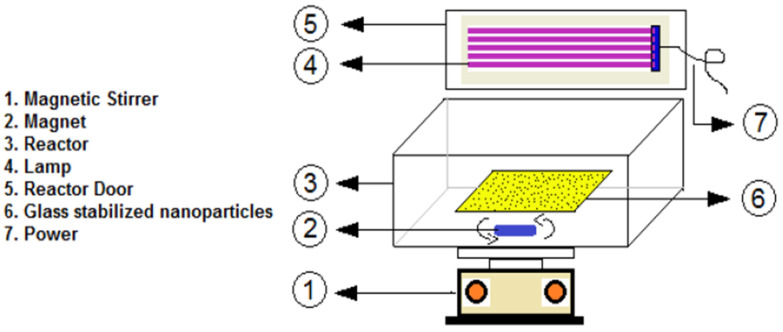
Schematic representation of the reactor used for the photocatalytic degradation of diazinon. Reprinted with permission from [151], 2020, Elsevier.

Additionally, diazinon degradation was achieved using ZnO NPs doped with various amounts (0.5, 1, and 2 mol%) of tungsten (W). The highest diazinon removal efficiency (99%) was observed with 2 mol% W-doped ZnO at neutral pH, with an initial diazinon concentration of 10 mg L^−1^ and a contact time of 180 min. Moreover, 2 mol% W-doped ZnO consumed less electrical energy during the degradation process compared to other photocatalysts (Figure 10) [151].

### 4.3. Optical and Electromagnetic Property-Based Applications

Technological advances have led to the widespread use of electronic communication devices, contributing to increased electromagnetic (EM) pollution. This pollution not only poses a significant threat to public health and the environment but also interferes with the operation of electrical devices. As a result, there is a growing demand for efficient EM radiation absorbers that are lightweight, possess significant absorption capacity, offer wide absorption bands, have potential engineering applications, and ensure environmental stability. In recent decades, researchers have identified graphene-based materials as ideal candidates for EM wave absorption due to their excellent properties [152,153,154,155,156].

One example of effective EM wave absorption is the work of Li et al., who synthesized ZnO NPs on mesoporous carbon hollow microspheres (PCHMs) [152]. The composite, created by combining paraffin with PHCM-deposited ZnO annealed at 700 °C, achieved a reflection loss (RL) of 12 dB over the entire X-band, surpassing the performance of previously reported ZnO-based materials. When the composite thickness was adjusted between 3.3 and 4.3 mm, the RL remained below −8 dB across the X-band, making it a highly efficient absorber.

In addition, ZnO NPs, with an average size of 15.8 nm, were synthesized using Zn(CH_3_COO)_2_·2H_2_O and urea, and characterized based on their light absorption properties and bandgap energy. The optical bandgap energy of the ZnO NPs was within an ideal range for visible light-driven photocatalysis and applications in solar cells and optical devices [72].

Further studies have explored the dielectric properties of colloidal ZnO nanospheres synthesized in two steps with the aid of a surfactant [157]. The nanospheres were composed of pure hexagonal ZnO. Dielectric studies conducted over a frequency range of 1 Hz to 1 MHz revealed that both the dielectric constant and dielectric loss decreased as frequency increased. Moreover, the electric modulus showed a significant peak shift to higher frequencies, implying a distribution in ionic relaxation times.

Another important study featured the development of a C/ZnO core/shell nanostructure, which efficiently attenuated EM radiation due to its surface functional groups, numerous interfaces, and excellent impedance-matching properties [158]. When combined with paraffin wax, the composite achieved a minimum reflection coefficient of 52 dB at 11 GHz for a sample thickness of 1.75 mm and a filler loading of 40%. At a 1:1 mass ratio, the composite’s reflection coefficient was 14.85 dB, with absorption being the primary mode of attenuation. This C/ZnO core–shell structure outperformed pure carbon spheres and ZnO hollow spheres in terms of dielectric loss, cost, and impedance matching, making it ideal for the design of high-performance EM wave absorbers.

Furthermore, vertically aligned ZnO NRAs have been fabricated for use in UV photodetectors (PDs) [154,159]. A thin layer of ZnO seeds connecting patterned Au/Ti electrodes was created using a shadow–mask approach, followed by vertical growth of ZnO NRAs in solution at 90 °C. This produced a metal–semiconductor–metal PD structure, and the effect of NRA size on photocurrent was studied by varying the growth solution concentration. The PD, with a channel width of 10 μm and ZnO NRAs grown at 25 mM, exhibited a photocurrent of 1.91 × 10^−4^ A at an applied bias of 10 V under 365-nm UV light irradiation. The PD performance improved further with a 15 μm channel width, yielding an excellent photocurrent on-off ratio of 37.4 and superior current transient characteristics. This development provides a cost-effective approach for the fabrication of UV PDs with high performance.

### 4.4. Microorganism Control

ZnO NPs exhibit strong antibacterial properties, making them promising candidates for blocking a variety of pathogenic agents. These properties are primarily attributed to their high specific surface area, which facilitates their interaction with bacterial cells, as well as the production of ROS such as superoxide anions, hydroxyl radicals, and hydrogen peroxide [160,161,162,163]. The antibacterial mechanism involves the accumulation of ZnO NPs on the outer membrane or within the cytoplasm of bacterial cells. This accumulation triggers the release of Zn^2+^ ions, leading to membrane disruption, protein degradation, and genetic instability, ultimately resulting in bacterial cell death [164].

**Figure 11:**
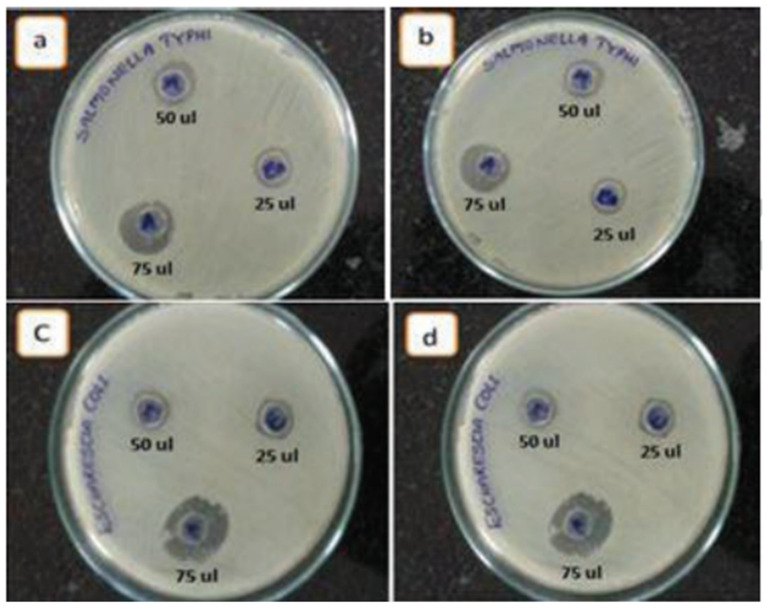
Antibacterial activity of ZnO NPs/FLG. Inhibition zones observed for (**a**,**b**) *S. typhi* and (**c**,**d**) *E. coli*. Reprinted with permission from [164], 2015, Elsevier.

The antibacterial activity of ZnO NPs has been evaluated using both Gram-negative and Gram-positive bacterial models, as outlined in the basic antibacterial testing method. In one study, ZnO NPs were decorated on few-layered graphene (FLG) sheets with ethylene glycol as a co-solvent and reducing agent to assess the composite’s activity against *E. coli* and *S. typhi* using the diffusion technique (Figure 11). The results indicated that *E. coli* was more susceptible to ZnO/FLG than *S. typhi* [164].

To further enhance antibacterial activity, Zn^2+^ ions were immobilized on the surface of amorphous SiO_2_ via hydrogen bonding with its hydroxyl groups. This process reduced the size of ZnO particles, preventing their agglomeration and ensuring a uniform distribution. The resulting ZnO-SiO_2_ composites exhibited superior antibacterial performance compared to pure ZnO, demonstrating a synergistic effect between the components [165].

**Figure 12:**
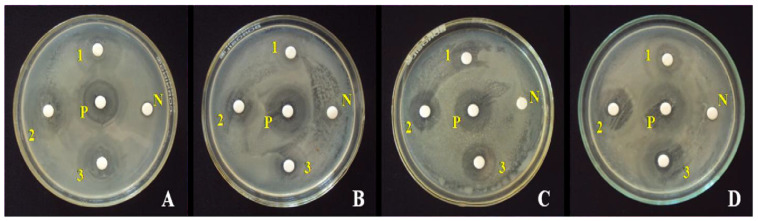
Activity of ZnO NPs synthesized from *C. candelabrum* against (**A**) *E. coli*, (**B**) *S. aureus*, (**C**) *B. subtilis*, and (**D**) *S. typhi*. N = negative control. P = positive control. Furthermore, 1, 2, and 3 denote ZnO NPs at loadings of 25, 50, and 100 μg disc^−1^, respectively. Reprinted with permission from [166], 2017, Elsevier.

ZnO NPs synthesized from *Ceropegia candelabrum* leaf extract and Zn(NO_3_)_2_ exhibited a hexagonal (wurtzite) phase with particle sizes ranging from 12–35 nm. These NPs demonstrated strong antibacterial activity against various bacteria, including *S. aureus*, *E. coli*, *B. subtilis*, and *S. typhi*, with the inhibition zone size ranking as *B. subtilis* < *E. coli* < *S. aureus* (Figure 12) [166]. Additionally, ZnO NPs synthesized using thyme (*Thymus vulgaris*) leaf extract, with particle sizes of 50–60 nm, showed higher antibacterial activity against Gram-negative bacteria compared to Gram-positive bacteria. Phenolic compounds in thyme extract, such as thymol and flavonoids, enhance membrane permeability by interacting with bacterial cell walls, promoting ion leaching, and potentially causing cell wall hydroxylation, which further damages bacterial integrity [167].

### 4.5. Solar Cells

Dye-sensitized solar cells (DSSCs) have garnered attention as promising third-generation solar cells due to their lower cost, relatively high efficiency, ease of fabrication, and flexibility compared to conventional Si-based counterparts [168,169,170]. However, charge recombination at the semiconductor–dye–electrolyte interface limits their performance.

Photoanode thickness significantly affects DSSC efficiency, with an optimal thickness of 40 µm yielding the highest efficiency [171,172,173,174]. ZnO NRs synthesized using Zn(CH_3_COO)_2_, ethylenediamine, and hydrazine have demonstrated enhanced performance as shell layers for TiO_2_ films. By varying the number of electrophoresis cycles, at optimized conditions, an efficiency improvement from 4.66% (unmodified TiO_2_) to 7.13% with two cycles.

**Figure 13:**
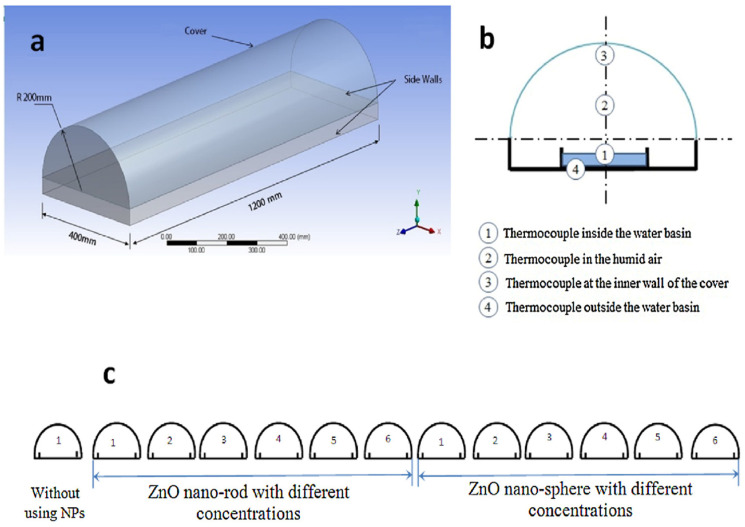
(**a**) Three-dimensional diagram of a half-tubular solar still, (**b**) schematic diagram of the half-tubular solar still with thermocouple locations, (**c**) arrangement of thirteen solar stills. Reprinted with permission from [176], 2017, Elsevier.

To further enhance ZnO nanostructures, Ga- and Al-co-doped ZnO (GAZO) thin films have been used as seed layers for ZnO NWs growth. Optimization of GAZO films by adjusting the temperature, growth period, and solution concentration yielded hexagonal ZnO NWs with high crystallinity, low electrical resistance (1.4 × 10^–2^ Ωcm), high transmittance (50–80%) [175]. Similarly, co-solvent-assisted methods have been explored to control ZnO morphology. For example, applying NR structures to solar still basins increased efficiency by 30–38% compared to nanospheres, owing to their smooth geometry and larger surface area (Figure 13) [176].

Again, ZnO–CdS nanocomposites synthesized with varying molar ratios (25:75, 50:50, 75:25) through hydrothermal methods showed dielectric properties influenced by synthesis conditions. Frequency-dependent AC, conductivity increases were attributed to defect-mediated hopping processes [177].

### 4.6. Nanofluids for Heat Transfer

ZnO NRs, synthesized via a hydrothermal method, were dispersed in ethylene glycol at varying loadings (0.1, 0.2, and 0.3 vol%) to formulate nanofluids (NFLs), with stability maximized at 0.1 vol% [178]. The heat transfer coefficient (HTC) of these NFLs increased significantly with higher ZnO loading, surpassing that of pure ethylene glycol by factors of 6, 10, and 12 at loadings of 0.1, 0.2, and 0.3 vol%, respectively. However, at higher loadings, the HTC did not scale linearly, likely due to local agglomeration during measurements.

Direct absorption solar collectors (DASCs), which show great potential for solar energy harvesting, can benefit from NFLs due to their tunable optical absorption properties. Advanced composites incorporating plasmonic Au NPs and hedgehog-like hierarchical ZnO NPs, synthesized via a hydrothermal method, were developed to enhance DASC performance [179]. Studies on the influence of working fluid temperature on heating and cooling efficiencies revealed that, compared to the base fluid, the optimal hierarchical-Au/oil NFL achieved a 240% improvement in photothermal conversion efficiency, highlighting its promise as a working fluid for DASCs.

### 4.7. Other Applications

#### 4.7.1. ZnO/Polymer Nanocomposites

Recently, researchers have focused on the potential of ZnO nanocomposites with various polymers. ZnO/polymer nanocomposites, with ZnO NRs (50–75 nm) embedded in the polymer matrix, exhibit superior thermal properties compared to pure polymers [180]. Studies using potentiodynamic polarization, linear polarization resistance, and electrochemical impedance spectroscopy revealed that these nanocomposites effectively inhibit mild steel corrosion in 5% HCl.

#### 4.7.2. ZnO/Activated Carbon Composites for Supercapacitors

ZnO composites have shown high effectiveness in supercapacitor applications. Using an in situ reaction process ZnO-based activated carbon composites were synthesized from Chlorella vulgaris and asphaltene via two-step pathways. The composites displayed diverse morphologies and specific surface areas, and at a current density of 0.5 A·g^−1^, the specific capacitance within the potential range of −1.0 to 0 V was found to be 155 F·g^−1^. The excellent capacitance retention over 1000 charge/discharge cycles arises as ZnO acts as electrically conductive pathways [181].

Additionally, a simple hydrothermal synthesis process at 140 °C for 10 h was used to combine ZnO with activated carbon to form a well-dispersed nanocomposite. The specific capacitance of ZnO/AC-based electrodes reached 298 F·g^−1^ at a current density of 1 A·g^−1^. At a power density of 1 kW·kg^−1^, the composite’s energy density was 48.39 Wh·kg^−1^, demonstrating significant performance improvement due to the synergistic effect of the electric double-layer charge storage mechanisms of AC and the faradaic storage mechanisms of ZnO [182].

#### 4.7.3. ZnO-Based Hybrid Supercapacitor Electrodes

Several advanced ZnO-based hybrid electrodes have been developed to enhance supercapacitor performance. Notably, a tin oxide–zinc oxide (SZ) nanocomposite with a unique flower-like morphology was synthesized using a one-step hydrothermal process. The SZ-10 electrode exhibited a high specific capacitance (Csp) of 548.56 F·g^−1^, and after 5000 cycles, it retained 88.2% of its capacitance. Additionally, at a current density of 1 A·g^−1^, the SZ-10 electrode demonstrated an energy density of 45.72 Wh·kg^−1^ and a power density of 406 W·kg^−1^, showing excellent performance [183].

Moreover, a hybrid structure of ZnO on reduced graphene oxide (GO) was synthesized under hydrothermal conditions. The ZnO/GO composite demonstrated superior capacitance performance compared to pure ZnO synthesized under the same conditions. High-crystallinity ZnO NRs clustered on the GO layers, forming flower-like nanostructures that significantly enhanced the electrochemical performance of the composite [184].

Table 2 presents a summary of various ZnO discussed and provides an overview of the representative reaction systems where they have been applied, including those not covered earlier.

## 5. Summary and Conclusions

This review summarizes the features of the hydrothermal technique for nanomaterials synthesis, and morphology control of ZnO nanomaterials synthesized through the hydrothermal method, highlighting their applications in many areas including sensing, photocatalysis, water splitting, microorganism control, pesticides removal, optical, electronic, nanofluids, solar cell and supercapacitors. This review also emphasizes the advantages of hydrothermal synthesis, notably the use of water as a solvent, which is inexpensive, readily available, and environmentally friendly. The hydrothermal method allows for synthesizing controlled sizes and shapes of ZnO NPs, NRs, NFs, NWs, nanoplates, and nanospheres each tailored for specific applications. Synthesis of small ZnO particles with narrow size distribution is possible. ZnO NMs obtained through hydrothermal route are highly pure and crystalline. According to the survey, ZnO particle aggregation occurs in some cases. The findings also present that hydrothermal synthesis is fast, versatile, and suitable for producing a wide range of materials, including ceramic oxides, bioceramics, thin films, vanadates, and garnets. The catalytic activity and other performance of bare ZnO can be improved by hybridizing it with other materials such as metal and non-metal doped ZnO NMs, and ZnO-based nanocomposites. Overall, nanoscale ZnO can be prepared cost-effectively by hydrothermal method which has a lot of possibilities of applications in many sectors. Despite the completion of numerous researches, still, the area remains a vibrant area of research and development. For instance, the use of non-aqueous starting materials is still limited, and such studies are in the early stages. To optimize hydrothermal synthesis and expand its applications, further research is needed into the physicochemical properties of hydrothermal devices, solvents, and mineralizers, along with a deeper understanding of the chemical reaction mechanisms involved. In future study, the use of effective surfactants, stabilizing agents and surface-modifying agents can lead to large-scale synthesis. Also, scaling up the synthesis method is another area of research for industrial production of nanomaterials commercially.

## 6. Future Directions in the Hydrothermal Synthesis of ZnO Nanomaterials

The prospects of ZnO-based NMs hold significant potential for advancing technology and addressing urgent global challenges. The hydrothermal synthesis method, which utilizes high-temperature and high-pressure aqueous environments, is expected to play a crucial role in precisely tailoring the properties of ZnO NMs, driving innovation across various industries.

In electronics and photonics, ZnO-based NMs promise the development of more efficient and high-performance devices. They can be applied in next-generation photovoltaic systems, enhancing the efficiency and affordability of solar panels. Additionally, these materials contribute to advanced LEDs, lasers, and displays in the optoelectronics sector. Their applications extend beyond electronics: ZnO NMs’ exceptional sensitivity to gases makes them valuable for gas sensors in environmental monitoring, industrial safety, and healthcare diagnostics. Moreover, their photocatalytic properties assist in water purification and wastewater treatment, offering sustainable solutions to the global water crisis.

In healthcare, ZnO NMs show promise in drug delivery systems, medical imaging, and cancer therapy. Their biocompatibility and controlled drug-release properties make them candidates for personalized medicine and targeted therapies. ZnO-based NMs also have the potential to enhance energy storage solutions, improving the performance of batteries and supercapacitors for longer-lasting, more powerful energy storage devices.

Despite these exciting prospects, challenges remain. Safety concerns and toxicity assessments for biomedical and consumer applications are crucial for the responsible use of ZnO NMs. Regulatory frameworks must evolve alongside rapid advancements in nanotechnology to protect users and the environment. In conclusion, hydrothermal synthesis techniques are poised to enable precise engineering of ZnO-based NMs, unlocking breakthroughs in electronics, energy, healthcare, and environmental sectors. These developments promise to foster sustainability and economic growth, establishing ZnO-based NMs as a central focus for future research and development.

## Figures and Tables

**Table 1:** Summary of hydrothermal synthesis conditions of the precursor type, temperature, reaction time, pH, and the morphology of ZnO-based NMs.

Precursor	Reaction Temp. (°C)	Reaction Time (h)	Morphology of Product	Reference
ZnCl_2_ (Merck) (pH = 5, 7, 12)	160	3	Rod-like ZnO and Cube-like ZnO	[80]
Zn(NO_3_)_2_·6H_2_O (98%) & C_6_H_12_N_4_ (99%) (Chongqing Chuandong Chemical Reagent Corp., Chongqing, China)	120	10	ZnO NPs, NRs, Mixture (NPs + NRs), NFs (Aggregation of NPs and NFs)	[81]
Zn(NO_3_)_2_ & C_6_H_12_N_4_ (1:1) (Sigma Aldrich, St. Louis, MO, USA)	60, 90, 120	3	ZnO NRs and microrice structured ZnO thin films	[82]
Zn(NO_3_)_2_ & C_6_H_12_N_4_ (1:1)	90	3	ZnO NRs	[83]
Zn(CH_3_COO)_2_·2H_2_O (Sharlau, Barcelona, Spain)	170	12	ZnO NRs	[84]
Zn(NO_3_)_2_·6H_2_O (Sigma Aldrich) (pH = 12)	100, 125, 150	2	ZnO NPs, NFs, NRs	[86]
	120	1,2,3		
Zn(CH_3_COO)_2_·2H_2_O (Sigma Aldrich)	170	5	ZnO NFs	[87]
Zn(CH_3_COO)_2_·2H_2_O (99.0%, AR)	160	24	Undoped ZnO and Zn_0.95_Fe_0.05−*x*_Ni_*x*_O (x = 0, 0.02 and 0.05)NRs	[88]
Zn(CH_3_COO)_2_·2H_2_O (Sharlau, Barcelona, Spain) & Ag(NO_3_)_2_ (Sigma Aldrich, Hamburg, Germany)	170	6	ZnO NRs and Ag-ZnO Nanocomposites	[89]
Equimolar	95	12	Zn_1−*x*_B_*x*_ O nanostructures	[90]
Graphite Powder (Sigma Aldrich) & Zn(NO_3_)_2_·6H_2_O	80, 90	10	Graphene/ZnO Nanocomposites	[91]
Zn(CH_3_COO)_2_·2H_2_O (99.0%) & CuCl_2_.2H_2_O (99.0%)	180	10	CuO, ZnO, CuO/ZnO Nanocomposites	[92]
(FeCl_3_∙6H_2_O, 97%), (ZnSO_4_∙7H_2_O, 99.9%) & (HAuCl_4_∙3H_2_O, 98%) (Sigma Aldrich)		15 min	Ternary α-Fe_2_O_3_-ZnO-Au Nanocomposites	[93]
Zn(CH3COO)_2_·2H_2_O (Sinopharm Chemical Regent Co., Ltd., Shanghai, China)	150 135	10 8	PPy/ZnO nanohybrids	[94]
ZnCl_2_ & (Ni(NO_3_)_2_.6H_2_O (Sigma Aldrich, Hamburg, Germany)	160	24	ZnO/Ni(OH)_2_ nanocomposites	[96]
(Zn(NO_3_)_2_·6H_2_O & HAuCl_4_. H_2_O (Sigma Aldrich, Hamburg, Germany)	87.5	8	Au@ZnO core@shell NPs	[98]
FeSO_4_·7H_2_O, Fe(NO_3_)_3_·9H_2_O & ZnCl_2_ (EMERCK, A.R. grade)	200	18	Fe_3_O_4_@ZnO core-shell NPs	[99]

**Table 2:** Applications of hydrothermally prepared ZnO-based NMs.

Catalyst	Role	Object of Performance Evaluation	Reference
Few-layered graphene/ZnO	Antibacterial	*E. coli, S. typhi*	[164]
ZnO NP–loaded PMAA-g-PA membranes	Antibacterial	*E. coli*	[185]
ZnO–SiO_2_	Antibacterial	*E. coli*	[165]
Bio-based ZnO	Antibacterial	*E. coli, R. rhodochrous,* *B. subtilis, V. cholera*	[186]
ZnO-Ag NPs	Antioxidant	DPPH assay	[187]
Rod-like ZnO	Photocatalyst	Methylene blue	[137]
N-doped ZnO	Photocatalyst	Methylene blue	[188]
ZnO-MoS_2_-rGO	Photocatalyst	Methylene blue	[133]
ZnO/clay	Photocatalyst	Methylene blue	[189]
ZnO/Zn-Sn oxide	Photocatalyst	Methylene blue	[190]
Flower-like ZnO	Photocatalyst	Methylene blue	[191]
ZnO/Sn_3_O_4_	Sensor	Gas sensor	[108]
MoS_2_-coated ZnO	Electrocatalyst	H_2_ evaluation	[192]
Al-doped ZnO	Sensor	Acetaldehyde	[109]
ZnO/activated carbon	Electrocatalyst	Supercapacitor	[181]
1D α-MoO_3_/0D ZnO	Sensor	Ethanol	[110]
Polypyrrole/ZnO	Sensor	Ammonia	[94]
Fe/Ni-co-doped ZnO	Sensor	Hexahydropyridine	[64]
